# Hair Evaluation Methods: Merits and Demerits

**DOI:** 10.4103/0974-7753.58553

**Published:** 2009

**Authors:** Rachita Dhurat, Punit Saraogi

**Affiliations:** Department of Dermatology, T.N.M. College and B.Y.L. Nair Ch. Hospital, Mumbai Central, Mumbai - 400 008, India

**Keywords:** Alopecia, diagnostic methods for hair loss, evaluating hair loss, hair loss, quantifying hair loss

## Abstract

Various methods are available for evaluation (for diagnosis and/or quantification) of a patient presenting with hair loss. Hair evaluation methods are grouped into three main categories: Non-invasive methods (e.g., questionnaire, daily hair counts, standardized wash test, 60-s hair count, global photographs, dermoscopy, hair weight, contrasting felt examination, phototrichogram, TrichoScan and polarizing and surface electron microscopy), semi-invasive methods (e.g., trichogram and unit area trichogram) and invasive methods (e.g., scalp biopsy). Any single method is neither 'ideal' nor feasible. However, when interpreted with caution, these are valuable tools for patient diagnosis and monitoring. Daily hair counts, wash test, etc. are good methods for primary evaluation of the patient and to get an approximate assessment of the amount of shedding. Some methods like global photography form an important part of any hair clinic. Analytical methods like phototrichogram are usually possible only in the setting of a clinical trial. Many of these methods (like the scalp biopsy) require expertise for both processing and interpreting. We reviewed the available literature in detail in light of merits and demerits of each method. A plethora of newer methods is being introduced, which are relevant to the cosmetic industry/research. Such methods as well as metabolic/hormonal evaluation are not included in this review.

## INTRODUCTION

Various methods are available for evaluation of a patient presenting with hair loss. Most of them do not fit the 'ideal' requirements to suit the needs of the clinician, researcher or the patient. However, most methods, when interpreted with caution, provide valuable insights into patient diagnosis and monitoring. The three-step approach to patient assessment includes a detailed history, clinical examination and investigations.

The parameters of hair growth that are of major interest to the clinician are diameter, density and hair cycle status.

Hair evaluation methods are grouped into three main categories:

### Non-invasive methods

e.g., Questionnaire, daily hair counts, standardized wash test, 60-s hair count, global photographs, dermoscopy, hair weight, contrasting felt examination, hair feathering test, phototrichogram and TrichoScan.

### Semi-invasive methods

e.g., Trichogram and unit area trichogram (UAT).

### Invasive methods

e.g., Scalp biopsy.

The following review would highlight the various available methods, with emphasis on the merits and demerits of each.

Readers are referred to further reading regarding hormonal and systemic evaluation in a patient with hair loss. Also, many investigations available today, such as confocal microscopy, scanning and transmitted electron microscopy, hair tensile strength, amino acid analysis, hair mineral analysis, atomic force microscopy, etc. are of interest from the view point of the cosmetic industry and research. These have not been included in this review.

## QUESTIONNAIRE

It consists of a set of questions for patient self-assessment, which have been shortlisted and psychometrically evaluated for validity.

### Male androgenetic alopecia

Merck Research Laboratories have developed a questionnaire for assessment of male AGA to supplement clinical measure in trials examining the effects of finasteride.

Patients assess their scalp hair by choosing the answer for four questions on treatment efficacy and three questions on satisfaction with appearance.[1]

These were:

Q1: Since the start of the study, I can see my bald spot getting smaller.

A: Strongly agree to strongly disagree

Q2: Because of the treatment I have received since the start of the study, the appearance of my hair is:

A: Lot better to a lot worse

Q3: Ever since the start of the study, how would you describe the growth of your hair?

A: Greatly increased to greatly decreased.

Q4: Ever since the start of the study, how effective do you think the treatment has been in slowing down your hair loss?

A: Very effective to not effective at all.

Q5: Compared to the beginning of the study, which statement best describes your satisfaction with the appearance of:

- The hairline front of your head?
- The hair on top of your head?
- your hair overall?

A: Very satisfied to very dissatisfied.

### Female AGA

The four-item Women's Hair Growth Questionnaire[2] is one such validated measure of perceived hair growth for females, which includes questions based on:

1. Growth of hair
2. Amount of noticeable new hair
3. Visibility of the scalp
4. Rate of hair loss

### Merits and demerits

These questionnaires are easy to comprehend and record. The scores have been shown to correlate with hair counts, although modestly. These have been used in clinical trials for the assessment of therapeutic response. But, these are subjective and can only be used as an adjunct in the assessment of response to therapy.

## DAILY HAIR COUNTS

**Figure 1:**
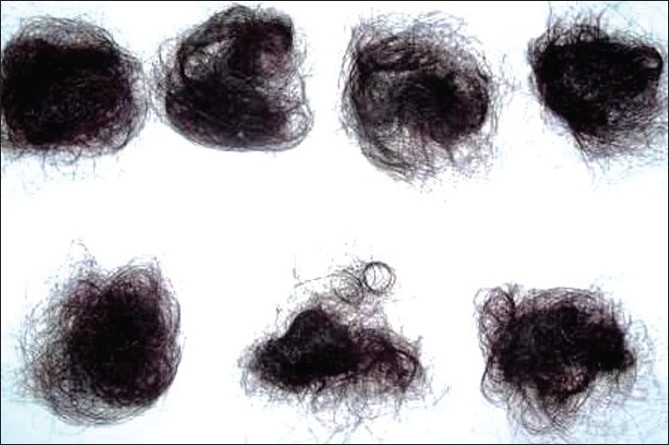
Daily hair count - collected hairs for 7 days

Daily scalp hair counts can be useful to the physician to help quantify how much the patient is losing and make sure that this is not more than the physiologic hair loss. It is said that it is normal to loose up to 100 hairs per day. Patients are instructed to collect hairs shed in one day, count them and place them in plastic bags. All shed hairs in the shower or sink or on the brush are collected. Daily hair counts for 7 days are maintained. It is expected to loose more hairs on shampoo days [Figure 1].[3,4]

If the patient is loosing more than 100 hairs per day, the hair should be examined microscopically to detect the pathology in hair bulb and hair shaft abnormalities. Appearance of the hair bulb can distinguish between telogen effluvium, anagen effluvium and active diffuse alopecia areata.[5]

## STANDARDISED WASH TEST

In the wash test, the patient refrains from shampooing for 5 days and then he/she shampoos and rinses the hair in the basin with the hole covered by gauze. The hairs remaining in the water and the gauze are collected and sent for examination. Hairs must be counted and divided into ≤3cm and ≥5 cm in length. This is an important technique to differentiate telogen effluvium from female-pattern hair loss. The 'modified hair wash test' demonstrates that in FAGA 58.9% of hair is vellus, whereas in chronic telogen effluvium (CTE), there are only 3.5%.[6]

## 60-S HAIR COUNT[7]

The technique comprises of the following four steps:

1. Before shampooing, comb your hair for 60 s over a pillow or sheet of contrasting color to your hair, starting with the comb at the back top of the scalp and moving the comb forward to the front of the scalp.
2. Repeat the procedure before three consecutive shampooings (e.g., if you shampoo every other day, then repeat the procedure every other day) and always use the same comb or brush.
3. Count the number of hairs in the comb or brush and on the pillow after each hair count and record.
4. Repeat the above procedure monthly and bring the results to your dermatologist.

### Merits and demerits (daily hair counts, wash test, 60-S hair counts)

Performing a hair count is tedious and time-consuming for the patient. But, it is something patients can do on their own and monitor their progress. The method is very subjective and it is usually difficult to come to a certain diagnosis. The number-100-is arbitrary. No clinical study or standardized method has validated the number 100. Whether 100 pertains to both men and women and whether or not the hair shedding changes with age has also not been established. In personal observation, various estimates for normal hair shedding have ranged from 10 to 250![7]

In a study of 404 females without hair or scalp disease, daily lost hair was collected over 6 weeks, where the mean hair loss rate was found to range from 28 to 35 per day.[8] Also, fewer than 50 hairs can be significantly abnormal in a patient having lost 50% of his/her hair volume.

Diagnostic definitions for cut-offs cannot be established for wash test as vellus hair count on the surface does not correlate with actual vellus hair counts (on scalp biopsy), as many vellus hair follicles fail to reach the scalp surface.

It is cumbersome for the patient to abstain from shampooing for 7 days.

We advice daily hair collection over 4 days after last shampooing in our center. Also, daily hair counts more than 70 are further probed into.

Falsely elevated numbers in 'daily hair counts' and the '60-s hair counts' tests could be due to hair breakage during combing.

These techniques, although easy to perform and inexpensive, are neither standardized nor diagnostic. They however give the clinician an estimate of the amount of hair shedding. Also, the 60-s hair counts cannot adequately measure the number of hairs shed from the lateral and posterior portions of the scalp.

## PULL TEST

This is also known as the 'traction test' or 'Sabouraud's sign' or the 'pull-out sign.'[9]

**Figure 2:**
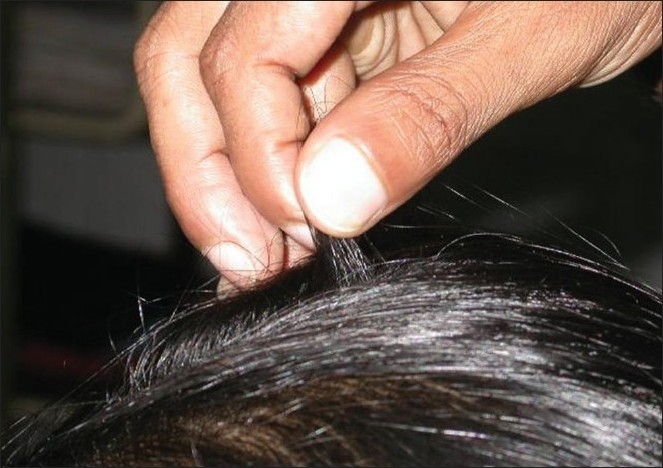
Pull test-approximately 60 hairs are grasped from the proximal portion of the scalp and tugged from the proximal to the distal end

Approximately 20-60 hairs are grasped between the thumb, index and middle fingers from the base of the hairs near the scalp and firmly, but not forcefully, tugged away from the scalp. If more than 10% hairs are pulled away from the scalp, this constitutes a positive pull test and implies active hair shedding. The patient must not shampoo for at least a day prior to the pull test [Figure 2].

### Merits and demerits

This test is based on the concept of 'gentle' pulling of the hair to bring about shedding of telogen hairs.[10] It helps to assess the severity and location of hair loss. The test is positive in cases of telogen effluvium, anagen effluvium, loose anagen syndrome, early cases of patterned alopecia and at the advancing edge of alopecia areata. In cases of acute telogen effluvium, the pull test is positive over the entire scalp whereas in cases of AGA, it could usually be positive over the area of thinning.

The extraction of anagen hairs with thickened root sheaths strongly suggests cicatricial alopecia, even if the pull test does not reveal increased hair loss. Therefore, even though only a few hairs may be extracted, the pull test is always regarded as pathological if anagen hairs are present.[11]

Hair pull tests vary from person to person. It is a very rough method and difficult to standardize as it is subject to so much interindividual variation among investigators. The pulling force is not distributed uniformly all over the whole bundle, which creates variation in the pulling force from one hair to another.[12] It is also difficult to approximate the number of hairs grasped, thereby leading to false inference of the test. Moreover, negative tests do not exclude the diagnosis.

## GLOBAL PHOTOGRAPHY

**Figure 3:**
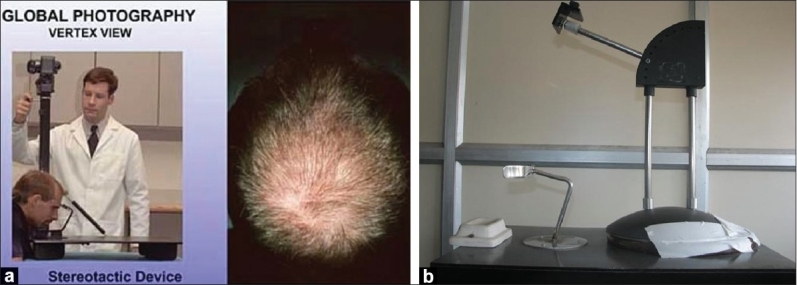
(a) Stereotactic device, (b) Stereotactic device-local modification

The Canfield technique has recently been validated. Use of a stereotactic positioning device on which the patient's chin and forehead are fixed, and on which a given camera and flash device are mounted, assures that the view, magnification and lighting are the same at consecutive study visits. It is important to ask the patients to keep the same hair style and color and the coordinators attempt to duplicate baseline hair parting and combing in subsequent follow-up visits. Four standard views (vertex, midline, frontal and temporal) are advocated [Figure 3a and b].[13]

### Merits and demerits

Paired comparison of global photographs (before and after treatment) is a more precise appreciation of hair growth after drug treatment compared with subjective evaluations of investigators and patients.

Standardized global photography with the use of the standardized seven-point rating scale (+3, +2, +1, 0, –1, –2, –3; greatly increased density to greatly decreased density) was used in a trial evaluating the efficacy of finasteride.[14]

Comparing of patient with an earlier photograph is better than comparing current hair loss with a memory of hair loss 3, 6 or more than 12 months ago.

## DERMOSCOPY AND VIDEODERMOSCOPY[15,16]

Unlike the conventional handheld dermoscope, videodermoscopy permits rapid, high-resolution viewing at several magnifications (up to ×1000 with advanced models), together with the ability to capture the viewed images digitally and to store them for later use.[17]

Images can usually be obtained with this system at 20×-70× magnifications. Dermoscopy and videodermoscopy have a role in the diagnostic assessment of scalp and hair disorders. Information may be used in conjunction with clinical and pathologic data to render a more accurate diagnosis. Few clinical features can be evaluated in greater detail with this technique than with the naked eye. These include epidermal and perifollicular scale, follicular ostia, hair shaft diversity, exclamation-point hair. etc.

### Patterns seen under dermoscopy

#### Cicatricial alopecias

**Figure 4:**
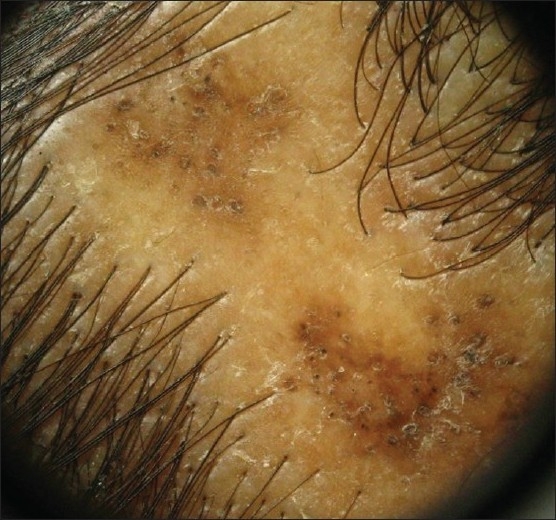
Dermoscope picture of cicatricial alopecia - features seen are hyperpigmentation, follicular plugging and scale crust

Erythema, scaling, perifollicular hyperkeratosis, atrophy, dyspigmentation, pustules or crusting can be seen in cicatritial alopecia. Irregularly paced hair shafts and hair tufting (dolls hair) are additional clues to a scarring process [Figure 4].

#### AGA

**Figure 5:**
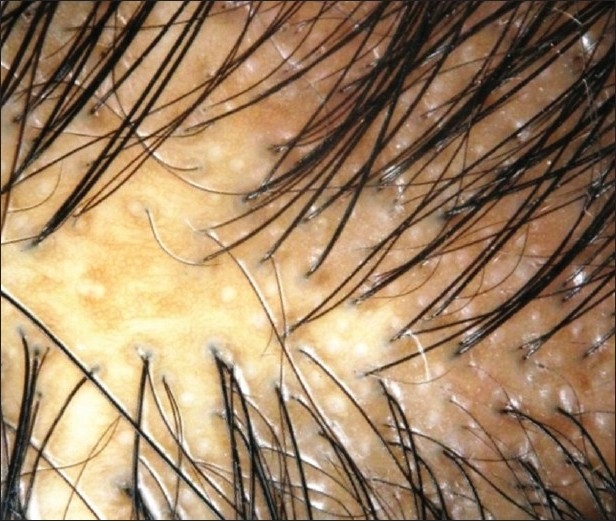
Dermoscope picture of FPHL - small bald areas representing exogen hair follicle

Dermoscopic features diagnostic of AGA are:

1. Hairs with a different caliber, reflecting progressive hair miniaturization
2. Brown halo, roughly 1 mm in diameter, at the follicular ostium around the emergent hair shaft
3. Small bald areas with numerous empty follicles -Exogen (eraser-like areas representing the delayed eruption of anagen hair after the telogen hair has been shed) [Figure 5]
4. Scalp pigmentation because of sun exposure

#### Yellow dots

The presence of yellow dots differentiates alopecia areata from trichotillomania or telogen effluvium. They are thought to represent degenerated follicular keratinocytes and sebum within the affected follicles. However, they are also seen in terminal hair bearing scalp, e.g., transitional zone of AGA and alopecia areata incognito.

#### Red dots

In early chronic cutaneous lupus erythematosus, dermoscopy shows a typical red dot pattern of follicular openings.

#### White dots with honeycomb pattern

**Figure 6:**
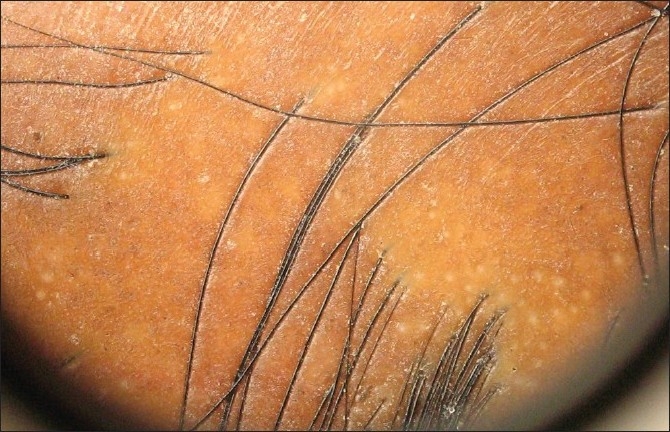
Dermoscope picture showing white dots

Numerous peripilar white dots that are varied in size could be appreciated at ×20 magnification in a few patients with lichen planopilaris or folliculitis decalvans, always in association with the honeycomb pigment pattern. These presumably mark the sites of targeted follicular destruction [Figure 6].

#### Merits and demerits

Dermatoscopy offers very fast and highly instructive clues to the diagnosis of hair and scalp disorders, including primary cicatricial alopecias.

Videodermoscopy also serves as a step prior to performing a biopsy. It can help the clinician to find the right place to take the sample, thereby avoiding unnecessary biopsies.

Predictive powers of patterns seen in dermoscopy with the clinical disease have yet to be quantified. Also, clinicians should be acquainted to identifying these patterns.

## HAIR WEIGHT

Hair weight determination requires that the hairs in a given target area be clipped close to the scalp at baseline, the hairs are allowed to grow for a fixed period of time and then the target-area hairs clipped again close to the scalp, collected and subsequently weighed.

### Merits and demerits

Change of weight from baseline as an endpoint in clinical trials of either a hair growth promoter or a hair growth inhibitor is useful as it simultaneously measures a potential change in hair count, width and length.

However, the methodology is difficult to control. To eliminate errors, careful degreasing, drying and control of humidity are essential. Standard clippers that fix the distance from the scalp must be used to avoid variations. It is difficult to precisely capture all hairs and only the hairs in a given target area. Also, because the measurement of hair weight is related to growth rate, each follow-up visit must be timed at exactly the same interval.

This method has been used only in clinical trials. However, currently hair weight evaluation is not recognized by the FDA as a primary end point in the evaluation of new drugs claiming hair growth promotion.[18,19]

## CONTRASTING FELT EXAMINATION

**Figure 7:**
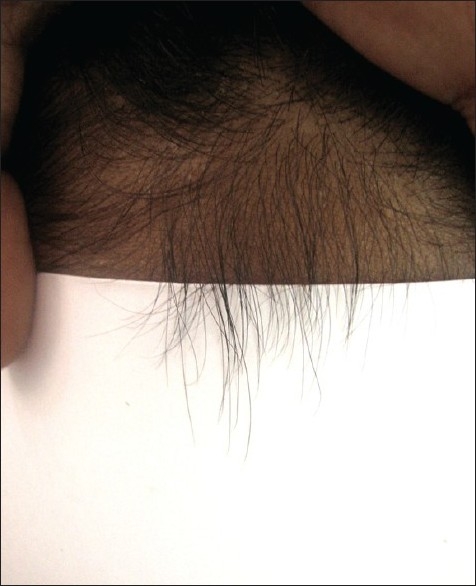
Contrasting felt examination-showing frontal fringe of vellus hairs

This test is used to see short, miniature hairs of the scalp. An index card with black felt glued on one side and white felt on the opposite side is used. After making a parting in the hair, the index card is held along the scalp. Fine short hairs with broken or tapered distal tips project up along the edges of the felt. These miniature hairs can be seen in the androgen-dependent areas of both men and women presenting with androgenetic alopecia. In a regrowing telogen effluvium, a classic short frontal fringe is seen [Figure 7].[5]

## HAIR PLUCK TEST/TRICHOGRAM[3]

**Figure 8:**
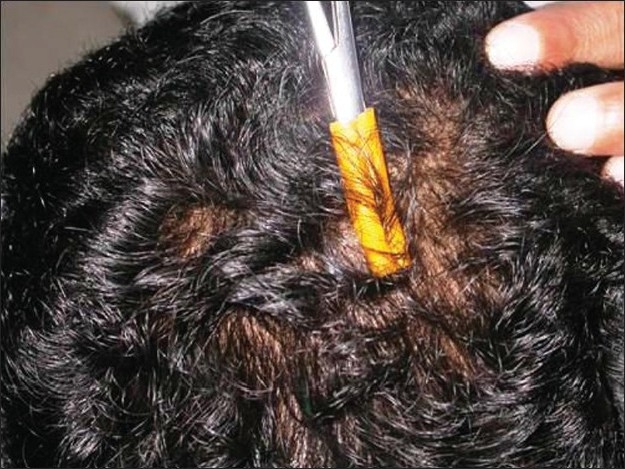
Trichogram - 60-80 hairs are grasped with a hemostat covered with rubber and are plucked, twisting and lifting the hair shafts rapidly in the direction of emergence from the scalp

**Figure 9:**
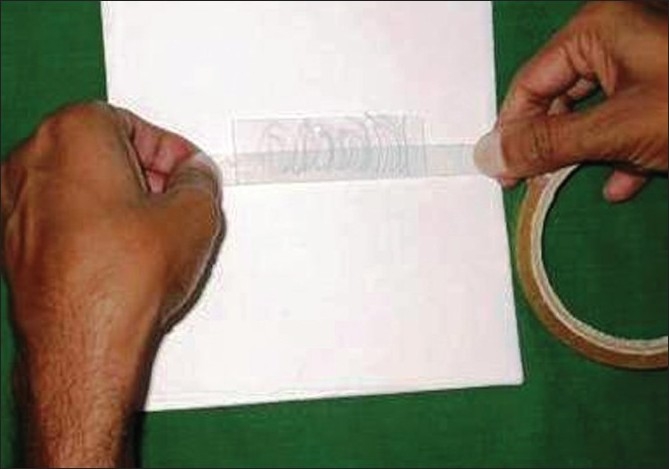
Preparing the trichogram slide - the plucked hairs are arranged side by side on a glass slide and taped

**Figure 10:**
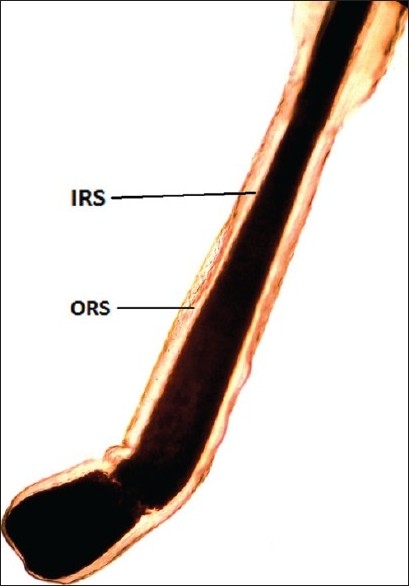
Anagen hair - forcibly plucked terminal anagen hair showing the pigmented bulb with 'hockey-stick' appearance (IRS: inner root sheath; ORS: outer root sheath)

**Figure 11:**
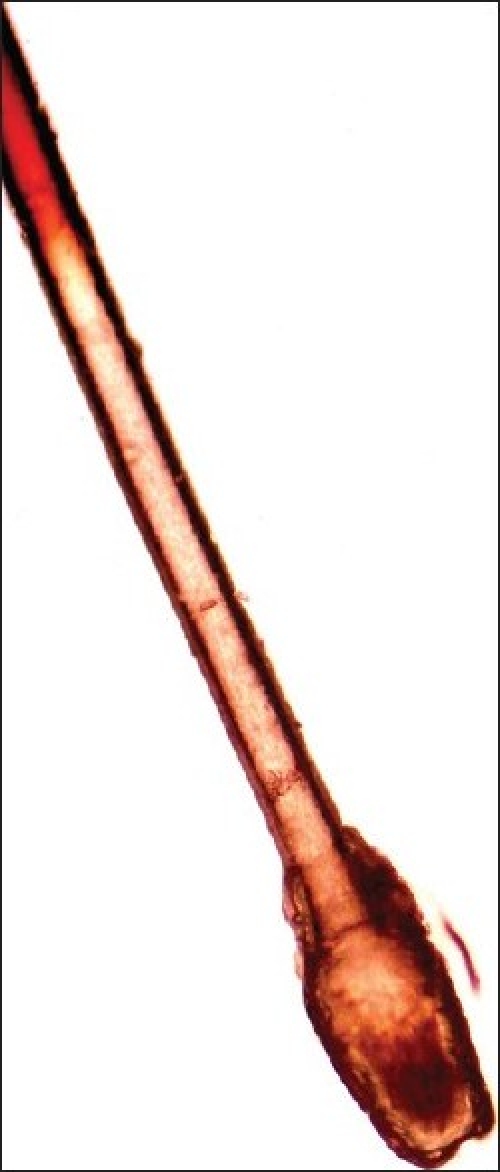
Telogen hair - forcibly plucked early telogen hair showing the hypopigmented, club-shaped cornified bulb with remanents of the cornified epithelial sac

To perform the pluck test, hairs are taken from specified sites on the fifth day after the last shampoo. The surrounding hairs are fixed with clips and 60-80 hairs are grasped with a hemostat covered with rubber. The hairs are plucked, twisting and lifting the hair shafts rapidly in the direction of immergence from the scalp. Hair shafts are then cut off 1 cm above the root sheaths and roots are arranged side by side on a slide and then taped. [Figures 8 and 9] The anagen hair bulbs are seen as darkly pigmented triangular or delta-shaped bulbs with an angle to the hair shaft and there is presence of inner root sheath [Figure 10]. The telogen hair is seen as less-pigmented hair with club-shaped hair bulb and there is absence of inner root sheath [Figure 11]. Anagen hairs are distinguished from telogen hairs and anagen to telogen ratios are calculated.

## UAT[20,21]

A fixed area is marked on the scalp through a template with a uniform fiber tip pen. All hairs within and on the scribed line were epilated individually with forceps/tweezers in the direction of the hair growth to minimize damage to the hair bulb.

### Merits and demerits

UAT is more accurate than the regular trichogram as it takes into account not only anagen/telogen ratios but also hair density and diameter.

The process of plucking is painful. UAT, although a meticulous technique, is quite laborious and requires special skill. The plucking procedure causes hair damage, leading to dystrophic hairs, which are difficult to interpret. Also, hairs with a clean transverse break in the shaft are seen when a deeply rooted anagen hair is plucked out. It is difficult to classify such 'snapped-off' hairs.

One more major demerit of the trichogram/UAT is that hairs in early anagen and vellus hairs are easily missed in a standard pluck because of their small size. Plucking is also known to change the course of the hair cycle.

Because of the relative values of anagen/telogen generated and the lack of exhaustive sampling, this technique is generally a poor indicator of disease activity and/or disease severity.

The UAT has been evaluated in terms of reproducibility and clinical relevance and have been used in few clinical trials.

## LIGHT MICROSCOPIC EXAMINATION

The hairs collected during pull test and pluck test are examined under the microscope. The hair shafts should be examined for fractures, irregularities, coiling and twisting or other hair shaft disorders. The free ends should be checked to see whether they are tapered, cut, fractured or weathered. If fungal infection is suspected, hairs should be placed on a glass slide in 20% KOH to demonstrate fungal hyphae and spores.

### Phototrichogram[22‐25]

#### Procedure

Saitoh introduced the phototrichogram in 1970. It is a non-invasive technique that allows *in vivo* study of physiology of the hair cycle and measurement of various hair growth variables.

These variables are:

1. Hair density
2. Hair thickness
3. Hair length
4. Linear growth rate

Various units are used to express these variables. These are hair density (number of hairs per cm^2^), thickness (micrometers), length (millimeter) and linear growth rate (millimeter per day). The variables are calculated on a specified area on the scalp, usually 1 cm^2^, over a specified time period, usually 2 days.

Thus, with the help of this procedure, the exact number of hairs per centimeter square, i.e. hair density, growing (anagen) and non-growing (telogen) hairs and hair diameter, can be derived and used to monitor the effect of treatment.

#### Clipping of hair

##### Day 0

The selected site is marked with the permanent marker using a 1 cm^2^ stencil. Then, hairs in the marked area were cut as close as possible to the scalp surface using a curved surgical scissor.

#### Hair diameter measurement

The clipped hairs are spread on a glass slide and dry mounted with a transparent adhesive tape to measure their diameter under the microscope using ×40 magnifications. A calibrated micrometer scale having a least measurement of 0.01 mm is used. The diameter of hairs is measured close to their bases using the measuring eyepiece.

#### Image recording

##### Day 0 (t0)

The marked area is then photographed using a digital camera, under fixed light conditions, from a fixed distance, using a fixed distance adapter specially designed for this purpose.

##### Day 2 (t2)

The patient is advised not to wash his/her hair for the next 2 days (to keep the permanent mark on the scalp intact) and then, exactly after 48 h, the second photograph was taken in a similar manner from the specified site.

##### Hair variables at t0

**Figure 12:**
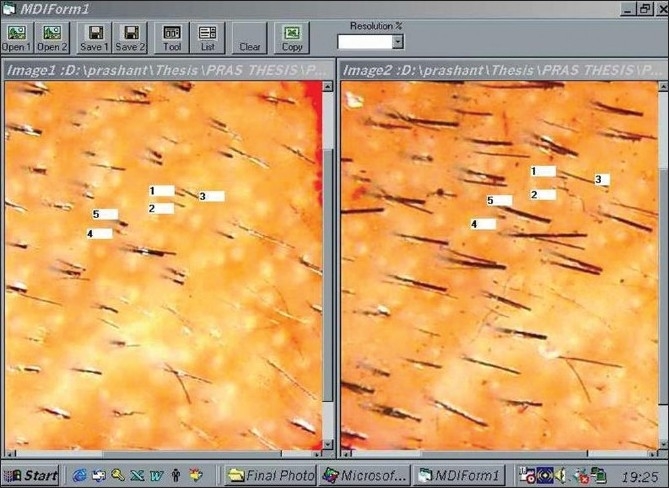
Phototrichogram - image analyser comparing day 0 and day 2 pictures from the same site

The following variables were evaluated from the first photograph taken at t0:

1. Density of hair in the specified area
2. Length of hairs at t0 (L1). (Even though hairs were cut very close to the scalp, remaining hair length could be measured with this software.) [Figure 12]

##### Hair variables at t2

The following variables were evaluated from the second photograph (t2):

1. The length of hairs at t2 (L2)
2. Hair growth in mm/day, (L2-L1)/2
3. Number of hairs showing hair growth in t2. (Anagen hairs)
4. Number of hairs not grown in t2. (Telogen hairs)
5. Anagen and telogen percentage

#### Contrast-enhanced phototrichogram[25]

The contrast-enhanced phototrichogram procedure involves coloring hair with black-colored dye immediately before starting the procedure. These temporarily colored hairs give a better contrast against the white scalp, making this method more sensitive for less-pigmented and thin hairs.

This contrast enhancement is not required in the Indian setting as we have usually darkly pigmented hairs, thus making the procedure still simpler for us to carry out.

### Merits and demerits

The phototrichogram is a non-invasive procedure, well tolerated by the patient. It is also possible to repeat the examination on the same area of the scalp at regular intervals, allowing evaluation of progress or reversion of pathology with treatment. This method has been validated with scalp biopsies. However, it is not diagnostic, is tedious and time consuming and subjective and requires expertise. Equipment and image analysis software is not easily available commercially.

### Scalp biopsy[26‐32]

#### Procedure

Scalp biopsies are indicated in all cases of cicatricial alopecia and undiagnosed cases of non-cicatricial alopecia.[28] The biopsies for non-cicatrizing alopecia are performed with transverse/horizontal sectioning rather than longitudinal/vertical sectioning. The transverse sectioning allows a greater number of hair follicles to be examined. According to Sinclair *et al*., the application of the diagnostic criteria achieved accurate diagnostic definition in 98% of women with triple horizontal biopsies vs. 79% with single horizontal biopsy.[32]

The biopsy procedure is performed under local anesthesia using lignocaine with 1:100,000 adrenaline and with a skin biopsy punch of at least 4 mm diameter, which gives an effective diameter of 12.6 mm^2^.[27] The biopsy must be deep and should include the entire follicular unit, including some subcutaneous fat. Usually, this involves a depth of 4 mm. In transverse sectioning, the biopsy specimen is bisected at level 1 mm below the dermoepidermal junction, which corresponds anatomically to the opening of sebaceous gland ducts into the follicle, where the numbers of vellus hair are maximum.[28‐30]

Normally, a scalp biopsy has 35-40 hairs at the upper level of papillary dermis and, at the reticular dermis level, the number is reduced to around 35 and, at the subcutaneous fat level, the numbers are 30. The upper level contains telogen, anagen as well as terminal, vellus and vellus-like miniaturized hairs. The deeper level contains anagen terminal hairs only.

The vellus follicles are defined as follicles containing hairs in which the diameter of the hair shaft is equal to or less than the thickness of the inner sheath of the same follicle and the diameter is ≤0.03 mm.[31]

The anagen hairs are identified in transverse sections by the presence of a normal-appearing inner root sheath and the absence of individual cell necrosis.

The catagen hair is a brief resting stage showing loss of matrix and thin epithelium of dermal papilla. The catagen hairs are counted along with telogen hairs.

The telogen hairs can be recognized below the level of the sebaceous duct by loss of inner root sheath. There is an irregular stellate configuration to the keratotic elements forming the remnants of the inner root sheath. Cornifying club having serrated rim, which interdigitates with surrounding outer root sheath, characterizes an early telogen hair bulb. Late telogen hair follicle shows completely cornified club.

The end stage of telogen or telogen germinal unit is seen as an irregular basaloid star-shaped island of cells marked by a peripheral nuclear palisade.

#### Histopathology of AGA

**Figure 13:**
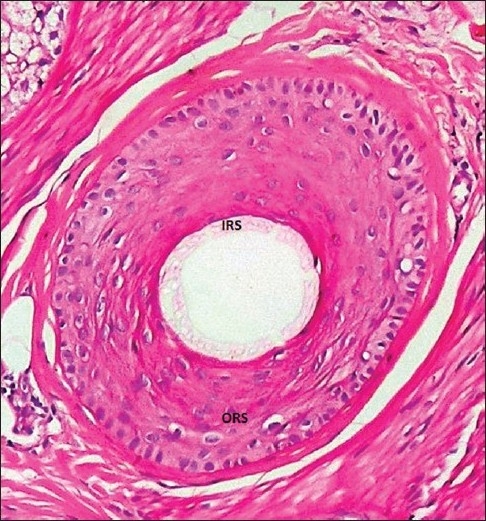
Terminal anagen hair-showing the inner root sheath and the outer root sheath (IRS, inner root sheath; ORS, outer root sheath)

**Figure 14:**
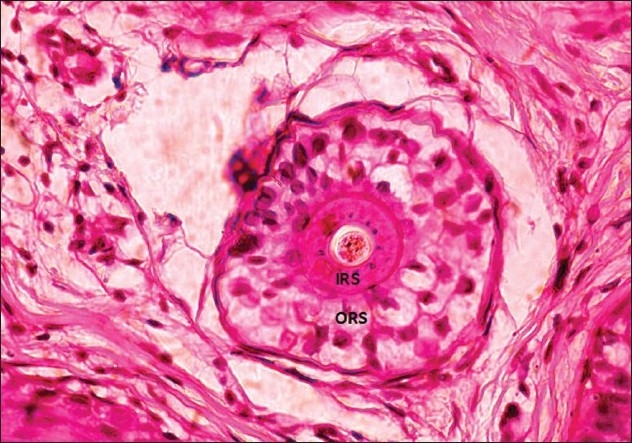
Vellus hair - inner root sheath thicker than the hair shaft

**Figure 15:**
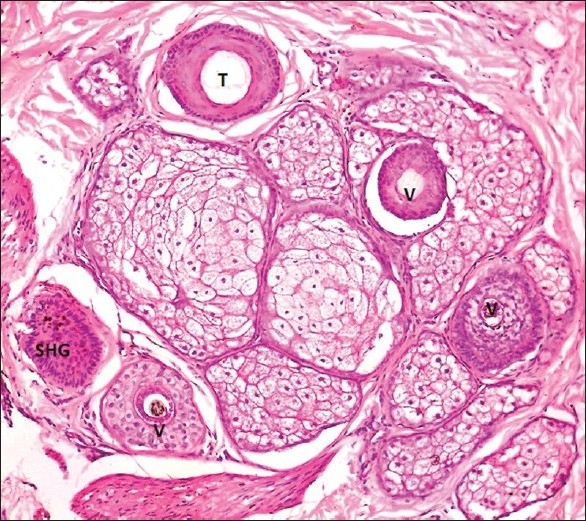
Androgenetic alopecia-scalp biopsy from the involved area from a male showing a follicular unit with three vellus hairs (V); one terminal (T) and one secondary hair germ (SHG)

AGA is characterized by progressive miniaturization of hair follicles. When the biopsy specimen is sectioned transversely at the level of opening of sebaceous ducts into the hair follicle, the hairs shafts appear vastly different in diameter. The position of the original terminal follicle is indicated by a follicular streamer (stellae or fibrous tract) extending from the subcutaneous tissue up to the course of the follicle to the miniaturized hair. Decreased terminal hairs and increased follicular streamers therefore characterize AGA. Sebaceous glands seem enlarged in relation to the miniaturized hair follicles. There is significant reduction in total follicular counts, measured by horizontal sectioning of scalp biopsy. The progressive reduction in the duration of anagen causes a relative increase in telogen hair [Figures 13‐15].

It was also reported that up to 40% of patients with AGA have perifollicular fibrosis, which is speculated to have an inverse relation with prognosis on treatment.[28,30]

The terminal to vellus ratio is normally 7:1. In AGA, the ratio is <4:1, which has been considered as diagnostic of AGA, whereas a ratio of >8:1 was considered diagnostic of CTE.

Between these two defined ratios are a number of equivocal cases, where the diagnosis remains unclear. Many of these unequivocal cases evolve into androgenetic alopecia with time.

#### Histopathology of telogen effluviu

**Figure 16:**
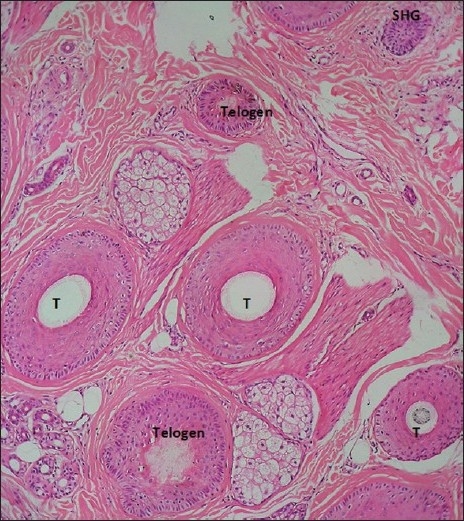
Scalp biopsy-showing telogen hairs, secondary hair germ (SHG) and terminal hairs (T)

The size of all the follicles is similar but there will be reduced number of terminal hairs when counted at the dermal-adipose tissue junction as compared with hairs counted at the upper dermis level. Total telogen hairs are counted and if the count is higher than 20%, it is suggestive of telogen effluvium. Because the period of telogen stage is short, it is difficult to see a high telogen percentage in all cases of TE [Figure 16].

### Merits and demerits

The scalp biopsy gives the actual number of hair follicles in the specified area, and their stage in the hair cycle can be assessed. It is also diagnostic in AGA and CTE.

Scalp biopsy was an important tool in diagnosing more than half (56%) of the cases of female pattern hair loss (FPHL), where women present with no obvious sparseness over the crown.[32]

Biopsies are also useful predictors of possible regrowth in long-standing alopecia areata.

Also, this is the only technique to diagnose cases of inflammatory alopecia associated with scarring.

A single 4-mm sample of scalp is not always adequately representative of the global process because of regional variation.

Also, scalp biopsy does not permit repeated sampling of a consistent area of scalp over time.

Scalp biopsy for the diagnosis of diffuse alopecia is a sensitive method, but it is painful and invasive. It is difficult for a patient suffering from diffuse alopecia to undergo biopsy unless he/she has enough motivation.

Multiple sections are required before commenting on the type of alopecia. This sectioning of biopsy specimen and then counting the number of follicles is a tedious job and requires expertise. The sections should always be oriented properly in a horizontal position rather than in an oblique position for correct analysis of histopathology and defining ratios.

For the diagnosis of AGA/CTE, the sectioning should be at the level of the sebaceous gland whereas for the diagnosis of acute telogen effluvium, the section has to be suprabulbar in the deep dermis so as to include maximum number of hairs in the telogen phase. Thus, for a given patient, numerous sections of the biopsy have to be performed.

### TrichoScan (TRICHOLOG GmbH)[33‐36]

**Figure 17:**
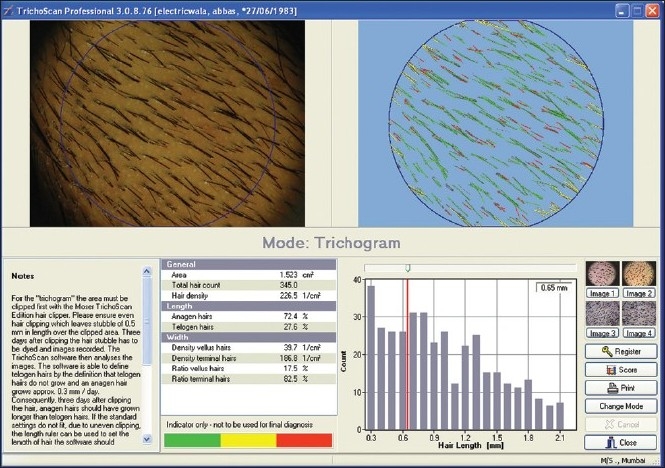
Trichoscan software interface

TrichoScan can be viewed as a modification of the classical trichogram. It combines standard epiluminescence microscopy with automatic digital image analysis for the measurement of human hair. The software quantifies the number of hairs and the anagen-telogen ratio within one operation. The use of TrichoScan initially involves shaving a scalp area (approx. 1.8 cm^2^). After 3 days, hairs in the shaven area are dyed and a digital photograph is taken at 20-fold magnification and saved. The TrichoScan software works on the basis that telogen hairs do not grow. The software uses this as a basis for calculation of the anagen-telogen ratio. Thus, the basic procedure is quite similar to that of the classical phototrichogram [Figure 17].

### Merits and demerits

The claimed advantage of this procedure lies in its simple and speedy photographic processing and the painlessness of the procedure with the reproducibility of results. However, there have been recent disputes regarding the accuracy of the TrichoScan.[34]

Also, in personal experience, we have observed that the TrichoScan software is error prone and not precise, detailed description of which is beyond the scope of this article.

#### Polarising light microscopy

Polarized light is the light in which all the rays vibrate in one plane. A polarizing microscope has two disk accessories and the placement of the discs is such that they allow light vibration in planes perpendicular to each other. Through the eyepieces, only a dark background is seen unless a doubly refractile object is placed in the path of polarized light, in which case the doubly refractile object appears illuminated against a dark background. It is a good tool for diagnosis in hair shaft disorders, e.g., 'tiger tail' appearance in trichothiodystrophy. Further reading is suggested for the use of polarizing microscopy.

## CONCLUSION

For decades, clinicians have examined methods of evaluating hair disorders and hair growth. With the developing market for newer drugs claiming hair growth-promoting benefits, there has been a greater need for reliable, economical and minimally invasive methods. In the above article, we have attempted to review the methods available and, moreover, feasible to the clinician. In our opinion, global photography and questionnaire are of greater significance to the individual hair clinician whereas in the various analytical methods available, the Phototrichogram is most suitable for clinical trials. Although scalp biopsy is diagnostic for female pattern hair loss, it is currently underutilized. While the results of fully automated computerized image analysis have largely been disappointing, there is a large scope of refinement this field.
